# Effects of riboflavin deficiency and high dietary fat on hepatic lipid accumulation: a synergetic action in the development of non-alcoholic fatty liver disease

**DOI:** 10.1186/s12986-023-00775-8

**Published:** 2024-01-02

**Authors:** Yanxian Wang, Xiangyu Bian, Min Wan, Weiyun Dong, Weina Gao, Zhanxin Yao, Changjiang Guo

**Affiliations:** Institute of Environmental and Operational Medicine, Tianjin, 300050 People’s Republic of China

**Keywords:** Riboflavin deficiency, Non-alcoholic fatty liver disease, Peroxisome proliferator-activated receptor gamma, Lipid metabolism, Oxidative stress

## Abstract

**Background:**

Non-alcoholic fatty liver disease (NAFLD) is characterized by excessive lipid accumulation in the liver. Riboflavin, one of water soluble vitamins, plays a role in lipid metabolism and antioxidant function. However, the effects of riboflavin deficiency on NAFLD development have not yet to be fully explored.

**Methods:**

In the present study, an animal model of NAFLD was induced by high fat diet feeding in mice and a cellular model of NAFLD was developed in HepG2 cells by palmitic acid (PA) exposure. The effects of riboflavin deficiency on lipid metabolism and antioxidant function were investigated both in vivo and in vitro. In addition, the possible role of peroxisome proliferator-activated receptor gamma (PPARγ) was studied in HepG2 cells using gene silencing technique.

**Results:**

The results showed that riboflavin deficiency led to hepatic lipid accumulation in mice fed high fat diet. The expressions of fatty acid synthase (FAS) and carnitine palmitoyltransferase 1 (CPT1) were up-regulated, whereas that of adipose triglyceride lipase (ATGL) down-regulated. Similar changes in response to riboflavin deficiency were demonstrated in HepG2 cells treated with PA. Factorial analysis revealed a significant interaction between riboflavin deficiency and high dietary fat or PA load in the development of NAFLD. Hepatic PPARγ expression was significantly upregulated in mice fed riboflavin deficient and high fat diet or in HepG2 cells treated with riboflavin deficiency and PA load. Knockdown of PPARγ gene resulted in a significant reduction of lipid accumulation in HepG2 cells exposed to riboflavin deficiency and PA load.

**Conclusions:**

There is a synergetic action between riboflavin deficiency and high dietary fat on the development of NAFLD, in which PPARγ may play an important role.

**Supplementary Information:**

The online version contains supplementary material available at 10.1186/s12986-023-00775-8.

## Introduction

Non-alcoholic fatty liver disease (NAFLD) refers to a pathological syndrome characterized by excessive intracellular fat deposition in the liver, including simple hepatic steatosis (SFL), nonalcoholic steatohepatitis (NASH), fibrosis, cirrhosis and related complications [1]. NAFLD has become one of the leading causes of chronic liver diseases worldwide and is closely associated with obesity, type 2 diabetes mellitus and metabolic syndrome [2]. Globally, it was estimated that the prevalence of NAFLD was as high as 34.2% [3]. Despite its high prevalence, the pathogenesis of NAFLD is not clearly elucidated. The “two-hit” hypothesis proposed by Day and James suggested that impaired lipid metabolism is the first hit that leads to hepatic steatosis. The second hit comprises oxidative stress and lipid peroxidation, resulting in the development of NASH, fibrosis and cirrhosis [4]. Later, the “multiple hit” hypothesis was raised by Buzzetti et al., in which it was proposed that multiple insults (including insulin resistance, inflammatory cytokines, nutritional imbalance, gut microbiota, genetic and epigenetic factors) acted together in contributing to the development of NAFLD in genetically predisposed subjects [5]. Currently, it is recognized that impaired lipid metabolism plays an initial role in the pathogenesis of NAFLD [6, 7]. Peroxisome proliferator-activated receptor gamma (PPARγ), a transcription factor that belongs to the family of PPARs nuclear receptors, is implicated in a wide range of biological processes, especially in regulating lipid metabolism [8–10]. Its expression was significantly upregulated in the liver of NAFLD mice and its knockdown led to attenuated hepatic lipid deposition [6–8], implying that it may play an important role in the development of NAFLD.

Numerous epidemiological and clinical studies have linked certain nutrients and dietary patterns to the occurrence of NAFLD [11–13]. The current data highlight the correlation between NAFLD and high intakes of saturated fat, red meats and fructose [14–16]. Riboflavin, a water-soluble vitamin, is mainly involved in energy metabolism in the forms of flavin mononucleotide (FMN) and flavin adenine dinucleotide (FAD). It also acts as a cofactor of glutathione reductase (GR) and is closely related to antioxidant function [17]. Recently, a dietary survey revealed that besides high dietary fat intake, insufficient riboflavin intake was prevalent in NASH patients [18]. Our previous rat experiment revealed that riboflavin deficiency led to a significant lipid accumulation in the liver [19]. Furthermore, a label-free proteomics study in HepG2 cells confirmed that riboflavin deficiency significantly affected NAFLD pathway based on KEGG pathway analysis [20], indicating that riboflavin deficiency may contribute to the development of NAFLD. Since riboflavin is an essential cofactor for several key enzymes in the lipid metabolism and antioxidant system, it is not surprising that riboflavin deficiency contributes to the pathogenesis of NAFLD, especially in the presence of high dietary fat intake. We hypothesize that riboflavin deficiency may synergize with high dietary fat to accelerate NAFLD development by compromising lipid metabolism and antioxidant function. To test this hypothesis, we explored the effects of riboflavin deficiency on hepatic lipid accumulation and antioxidant function in mice fed a high-fat diet. An in vitro experiment was also carried out in HepG2 cells to validate the synergetic action of riboflavin deficiency and palmitic acid (PA) treatment on lipid accumulation and antioxidant function. The possible role played by PPARγ was further investigated.

## Materials and methods

### Chemical reagents

Riboflavin (purity ≥ 99%), dimethyl sulfoxide (DMSO), bovine serum albumin (BSA) and PA were obtained from Sigma-Aldrich Co. (St. Louis, MO, USA). The stock solution of riboflavin (5 mM) was prepared in DMSO. PA was dissolved in a 100 mM sodium hydroxide solution by heating at 70 °C for 30 min and then mixed with 10% fatty acid-free BSA solution at 37 °C for 1 h, to yield a 5 mM PA solution [21]. Cell counting kit-8 (CCK8) and TRIzol RNA extraction reagents were purchased from Invigentech (CA, USA). The protein quantification kit (BCA assay) and Oil red O staining kit were obtained from Beyotime Biotechnology Co., Shanghai, China. The assay kits for alanine aminotransferase (ALT), aspartate aminotransferase (AST), triglycerides (TG), total cholesterol (TC), glutathione reductase (GR), superoxide dismutase (SOD) and glutathione peroxidase (GSH-Px) were from Nanjing Jiancheng Bioengineering Institute, Nanjing, China. Fatty acid synthase (FAS), adipose triglyceride lipase (ATGL) and carnitine palmitoyltransferase 1 (CPT1) ELISA kits were from Jianglai Biotechnology Co., Shanghai, China. The primary antibodies used in this experiment were PPARγ (Abcam, Cat# ab178860), glyceraldehyde-3-phosphate dehydrogenase (GAPDH) (Bioworld, Cat# MB001). The secondary antibodies were goat anti-mouse IgG (H&L)-HRP (Bioworld, Cat# BS12478) and goat anti-rabbit IgG (H&L)-HRP (Bioworld, Cat# BS13278).

### Animal experiment

**Fig. 1:**
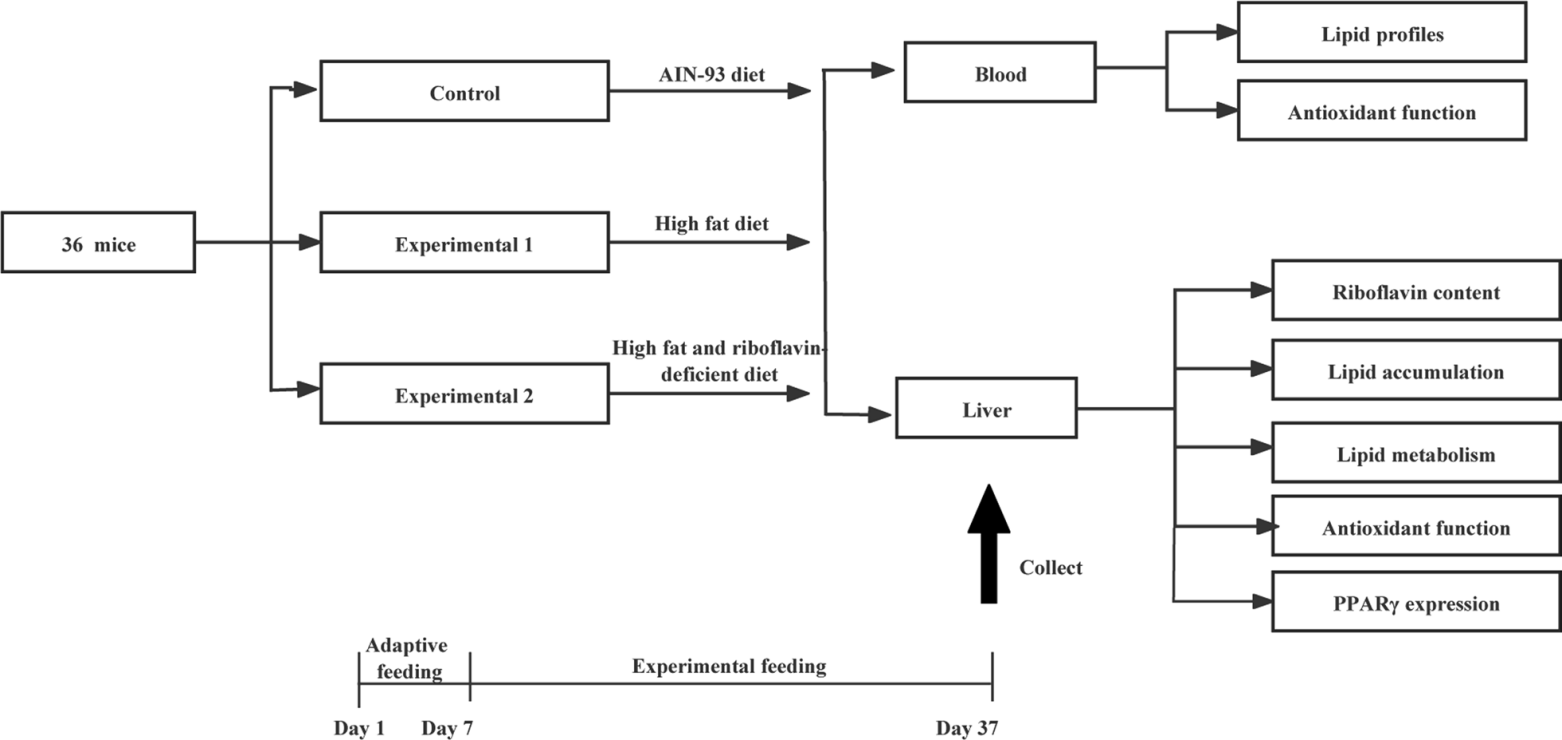
The protocol of animal experiment. C57BL/6 were randomly divided into three groups, including control (C), high fat diet (HFD) and high fat and riboflavin deficient diet (HFRD) groups, and fed for 30 days. After the feeding period, serum and liver samples were collected for follow-up analysis

Animal experiment was carried out by following current Chinese legislation on the care and use of laboratory animals. The experimental protocol was approved by the Ethical Committee of the Department of Scientific Management of the institute. Thirty-six male 6–8 weeks old C57BL/6 mice (obtained from Huafukang Biotechnology Co., Beijing, China) were housed in a temperature (23–26 °C) and humidity (40–60%) controlled room with a 12 h light/dark cycle. After 1 week of adaptive feeding on a regular AIN-93 M diet, the animals were randomly divided into three groups. The control group (C) was continuously fed the regular AIN-93 diet. The high fat diet group (HFD) was switched to a high fat diet containing 42% of energy from lard and 0.2% cholesterol [22], whereas the high fat and riboflavin deficient diet (HFRD) group to the high fat diet free of riboflavin, which was formulated according to the method of Bian et al. [19]. The compositions of the three diets were listed in Additional file 1: Tables S1–S3. The feeding period lasted 30 days. During the experiment, all mice had free access to water and food. Food intake was recorded daily, and body weight measured every 5 days. At the end of the experiment, mice were sacrificed under anesthesia via cervical dislocation. The blood collected from the orbital plexus was centrifuged to obtain serum. Liver tissues were harvested, fixed in 4% formalin at 4 °C for 24 h and then embedded in paraffin. Part of the liver samples were rapidly frozen in liquid nitrogen at − 80 °C for biochemical assays, ELISA and Western blot analysis. The chart of experimental protocol was shown in Fig. 1.

### Cell culture

Human hepatoma cell line HepG2 cells (purchased from Shanghai Institutes for Biological Sciences, Shanghai, China) were first grown to 60–80% confluence in Dulbecco’s modified Eagle’s medium (DMEM, Gibco) supplemented with 10% fetal bovine serum (FBS, Gibco) and antibiotics (100 U/mL penicillin and 100 mg/mL streptomycin) in an incubator (5% CO_2_) at 37 °C. Then, they were divided into the normal control and experimental groups. The control cells were continuously cultured in the DMEM medium. The experimental cells were transferred to the DMEM media (Basalmedia, Shanghai, China) formulated with different concentrations of riboflavin (0, 3, 12, 24 nM) in the presence or absence of PA. After cultured further for 96 h, cells were harvested and subjected to further analysis.

In our preliminary experiments, PA was selected to successfully induce lipid accumulation in HepG2 cells at the concentration of 10 μM without significantly influencing the viability of the cells. Therefore, the concentration of PA was set at 10 μM in this experiment. The concentrations of riboflavin in the medium were chosen based on the following reasons: 0 nM represents severe deficiency, which may occur in the plasma of premature infants treated with light therapy [23] and in cystic fibrosis patients with a severe deficiency [24]; 3 nM represents the level of riboflavin in the plasma of moderately deficient pregnant women [25]; 12 nM represents the riboflavin concentration in normal human plasma [26]; 24 nM represents a sufficient concentration of riboflavin in well-nourished human plasma [20].

### Hematoxylin and eosin staining and NAFLD activity score of the liver

The liver sections were processed according to the standardized hematoxylin and eosin (H&E) staining procedure. The pathological changes were observed under a light microscope and analyzed using the NAFLD activity score (NAS) method developed by the International Pathology Committee [27].

### Cell viability assay

After cells were washed with PBS, 10 μL CCK8 reagent was added and further incubated for 1 h at 37 °C with 5% CO_2_. The absorbance was measured at 450 nm using a microplate reader (BioTek, USA). The manufacturer’s instructions attached were strictly followed.

### Oil red O staining

The liver tissues were fixed in 4% formalin and embedded in paraffin wax. Then, they were embedded in optimum cutting temperature compound and stained with Oil red O kit according to the manufacturer's instructions [28].

The HepG2 cell samples were washed twice with PBS and then fixed with 4% polyformaldehyde for 10 min. Oil red O staining is performed according to the manufacturer's instructions. After dyeing, the cells were washed twice with PBS and observed under light microscope. Finally, the Oil red O stained in the cells was dissolved in 500 μL isopropanol, and 100 μL of isopropanol from each well was transferred to a 96-well plate. The absorbance at 510 nm was measured using a microplate reader to qualify the accumulation of lipid drops.

### Assessment of riboflavin status, liver function and lipid profiles

The contents of riboflavin, FMN and FAD in the liver were determined by a high performance liquid chromatography procedure described by Bian et al. [19]. The activities of ALT and AST in the serum, as well as the contents of TG and TC in the serum and liver were measured using the corresponding assay kits. All measurements were performed strictly in line with the instructions attached.

### Measurement of GR, SOD and GSH-Px activities

Corresponding commercial assay kits were used to measure GR, SOD and GSH-Px activities in the liver, serum or cultured cells. The activities GR, SOD and GSH-Px in the liver and cultured cells were normalized by the protein content as determined by BCA assay.

### Determination of ATGL, FAS and CPT1 levels

The protein levels of the enzymes involved in lipid metabolism in the liver and cultured cells, including ATGL, FAS and CPT1, were determined using corresponding commercial ELISA kits. All results were normalized by the protein content as determined by BCA assay.

### Western blot analysis

Total protein extracts of the cell lysates were prepared using RIPA lysis buffer and denatured at 95 °C for 10 min. Then, they were separated on 12% SDS-PAGE and electrophoretically transferred onto a PVDF membrane (Merck Millipore, NJ, USA). The immunoreactive protein bands were visible after interaction with the primary antibodies, followed by incubation with the corresponding secondary antibodies. GAPDH was used as the reference protein. Densitometric analysis was performed using a chemiluminescence scanner (Amersham Pharmacia Biotech, Inc., USA), and the results were expressed as the ratio of the optical density of the target proteins to that of GAPDH.

### Reverse transcription-polymerase chain reaction (RT-PCR) analysis

The mRNA expression of PPARγ was measured by RT-PCR analysis. The primer was purchased from Sangon Biotech Co., Ltd (Shanghai, China). Total RNA was extracted from the cultured cells using Trizol reagent and quantified using a Nano Photometer (Thermo Scientific, MA, USA). PrimeScript™ RT Master Mix (Takara, Beijing, China) was used to reverse-transcribe 400 ng of RNA into cDNA. The RT-PCR system consisted of 10 μL TB Green Premix Ex Taq II (Takara, Beijing, China), 0.8 μL upstream and downstream primers, 0.4 μL ROX Reference Dye II (Takara, Beijing, China), 2 μL template cDNA, and 6 μL DEPC water (Takara, Beijing, China). The reaction conditions were 95 °C for 30 s, 40 cycles of 95 °C for 5 s and 60 °C for 30 s. The expression level of the PPARγ gene was calculated by the 2^−△△Ct^ method using the housekeeping gene 18S rRNA as the reference. The primer sequences are listed in Table 1.

**Table 1:** Primer sequences used for RT-PCR analysis

	Forward primer (5′–3′)	Reverse primer (5′–3′)
PPARγ	GTGAAGGGCAAGCCACTCTG	AGAGAGGGTCCCATTTCCGA
18S rRNA	CAGCCACCCGAGATTGAGCA	TAGTAGCGACGGGCGGTGTG

### Small interfering RNA (siRNA) transfection

Regarding RNA silencing, the siRNA sequence targeting human PPARγ was designed and synthesized by Hanbio Technology Co., Ltd. (Shanghai, China). The specific sense and antisense strand sequences are shown in Table 2. HepG2 cells were transfected with 30 pmol siRNA for gene silencing using Lipofectamine RNAiMAX transfection reagent (Invitrogen, USA) according to the manufacturer’s instructions. Six hours after transfection, the transfection efficiency was observed by fluorescence microscopy. In addition, RT-PCR and Western blot were used to evaluate the transfection efficiency at the end of the cell culture.

**Table 2:** siRNA sequences for transfection

	Forward primer (5′–3′)	Reverse primer (5′–3′)
siRNA PPARγ	AUGGAGUCCACGAGAUCAUUUTT	AAAUGAUCUCGUGGACUCCAUTT
siRNA Control	UUCUCCGAACGUGUCACGUTT	ACGUGACACGUUCGGAGAATT

To investigate the role of PPARγ in lipid metabolism of HepG2 cells treated with riboflavin deficiency combined with PA load, PPARγ siRNA was transfected before being cultured in media containing 10 μM PA or/and riboflavin deficiency for 96 h. Finally, cells were collected and subjected to further analysis.

### Statistical analysis

All statistical analyzes were performed using SPSS version 21 software. Data are expressed as mean ± standard deviation. Differences among groups were analyzed by one-way analysis of variance followed by Tukey's multiple comparison test. Factorial analysis was applied to analyze the interaction between riboflavin deficiency and PA load. *P* < 0.05 was considered significant statistically.

## Results

### Animal experiment

#### Riboflavin deficiency is developed in mice after feeding the high fat and riboflavin deficient diet

**Fig. 2:**
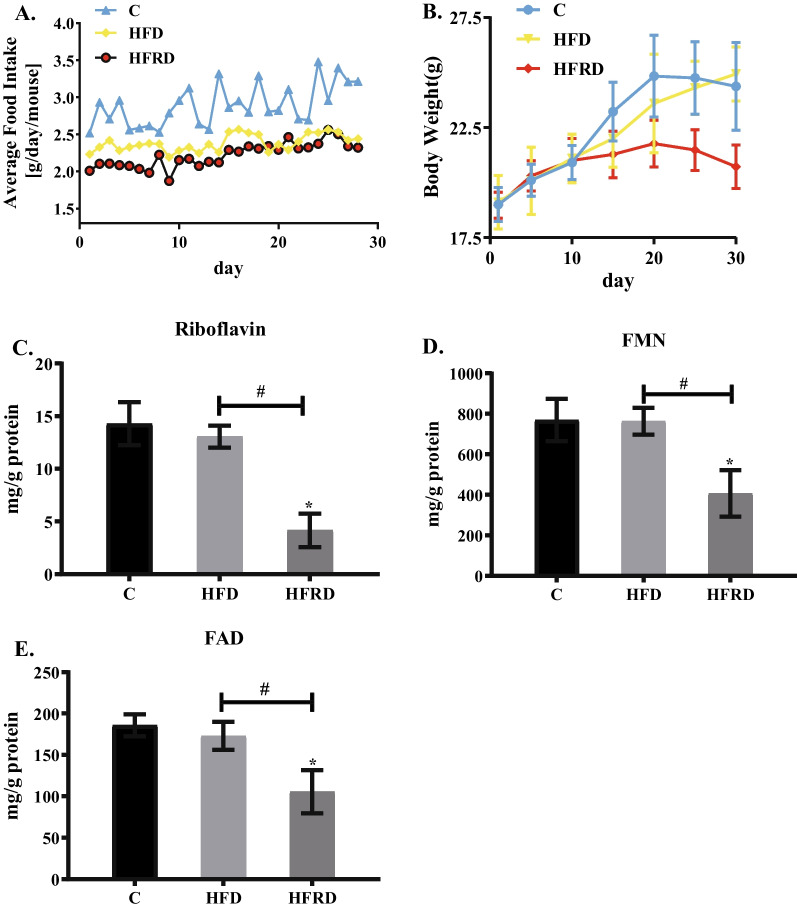
Changes of riboflavin status after feeding different diets in mice. C57BL/6 mice were randomly divided into three groups, including control (C), high fat diet (HFD), high fat and riboflavin deficient diet (HFRD), and fed for 30 days. **A** Changes of daily food intake. **B** Changes of body weight. **C**–**E** The contents of riboflavin, FMN and FAD in the liver as determined by HPLC. Data are expressed as means ± SD (n = 12). *P < 0.05 versus C; #*P* < 0.05 versus HFD

Compared to the C group, the HFD and HFRD groups consumed significantly less food during the experimental period (Fig. 2A). At the end of the experiment, body weight was significantly lower in the HFRD group than in the C and HFD groups (Fig. 2B). As shown in Fig. 2C–E, hepatic contents of riboflavin, FAD, and FMN in HFRD mice were significantly decreased by 70.8%, 43.2% and 47.15%, respectively in comparison with the C mice. However, there was no significant difference between the C and HFD groups. These results showed that riboflavin deficiency was successfully developed in HFRD mice.

#### Riboflavin deficiency further compromises lipid metabolism in mice fed the high fat diet

**Fig. 3:**
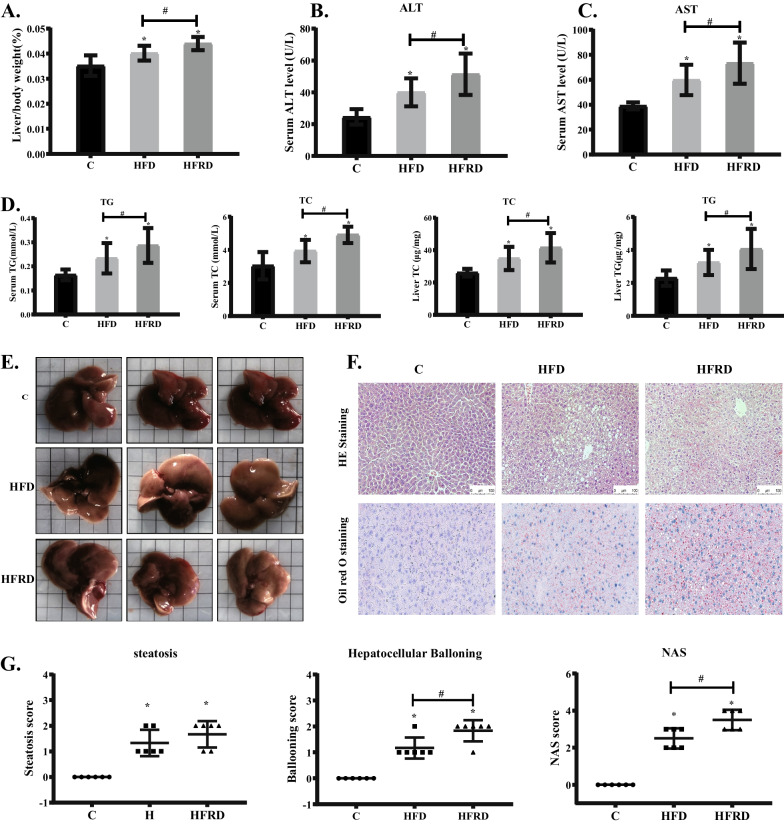
Changes of liver index, histopathological structure and NAS after feeding different diets in mice. C57BL/6 mice were randomly divided into three groups, including control (C), high fat diet (HFD), high fat and riboflavin deficient diet (HFRD), and fed for 30 days. **A** Liver weight as a percentage of body weight. **B**–**C** Serum ALT and AST levels were determined using corresponding kits. **D** Serum and liver TG and TC levels were determined using corresponding kits. **E** Anatomical view of the liver. **F** Representative pictures of HE (20×) and Oil Red O (40×) stained liver sections. **G** NAFLD activity score, 6 mice per group. Data are expressed as means ± SD. *P < 0.05 versus C; #*P* < 0.05 versus HFD

The liver index was remarkably increased in the HFD group compared to the C group. A more significant increase in the liver index were noted in the HFRD group (Fig. 3A). Meanwhile, serum activities of ALT and AST were significantly increased in the HFD group. Compared to the HFD group, a more significant increase in serum activities of ALT and AST were seen in the HFRD group (Fig. 3B–D), suggesting that riboflavin deficiency leads to a more severe liver damage in mice fed the high fat diet. In consistent with the changes in the liver index and serum activities of ALT and AST, similar changes were found in the liver and serum contents of TG and TC in the HFRD group, indicating that lipid metabolism is disturbed more significantly in mice fed the high fat and riboflavin deficient diet.

Macroscopic examination revealed that the livers became swollen and dull yellow in color after high fat diet feeding (Fig. 3E). Histopathological examination after H&E staining found that high fat diet feeding led to steatosis and balloon-like lesions in the liver (Fig. 3F) and NAS was significantly increased. The results of Oil red O staining showed that the accumulation of hepatic lipid droplets was remarkably increased in the HFD group. These pathological changes were worsen by riboflavin deficiency, as demonstrated by more significantly increased steatosis, ballooning and NAS in the HFRD group (Fig. 3F–G).

**Fig. 4:**
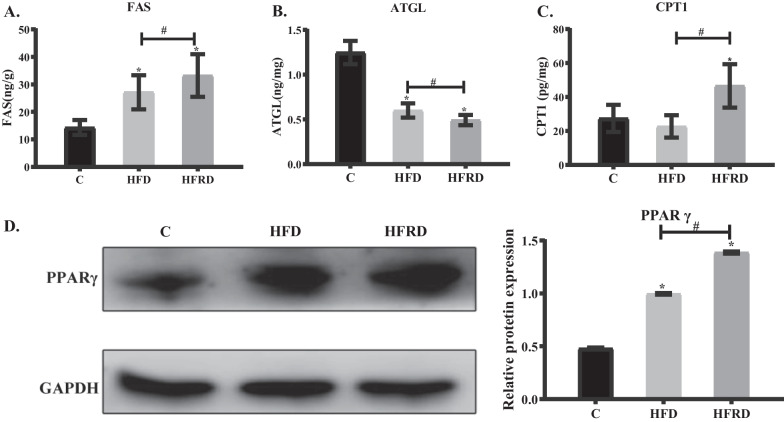
Expressions of hepatic FAS, ATGL, CPT1 and PPARγ after feeding different diets in mice. C57BL/6 mice were randomly divided into three groups, control (C), high fat diet (HFD), high fat and riboflavin deficient diet (HFRD), and fed for 30 days. **A**–**C** Liver FAS, ATGL and CPT1 contents were determined by the ELISA method. **D** Hepatic PPARγ and GAPDH protein expressions were measured by Western blot analysis. The histogram is the grey value analysis of the corresponding protein bands. Data are expressed as means ± SD (n = 3). *P < 0.05 versus C; #*P* < 0.05 versus HFD

The protein expressions of FAS, ATGL and CPT1, three critical enzymes in fatty acid synthesis, triglyceride hydrolysis, and fatty acid oxidation were detected in the liver using ELISA technique [29–31]. Compared to the C group, the expression of FAS in the liver of the HFD mice was significantly increased and further elevated by 22.5% in the liver of the HFRD mice (Fig. 4A). On the other hand, the expression of ATGL in the liver of the HFD mice was significantly decreased and further dropped by 18.3% in the liver of the HFRD mice (Fig. 4B). The content of CPT1 in the liver of HFD mice did not change significantly, while it was increased by 69.5% in the HFRD mice (Fig. 4C). These results showed that riboflavin deficiency resulted in increased fatty acid synthesis and decreased triglyceride hydrolysis in mice fed the high fat diet.

The expression of hepatic PPARγ was significantly up-regulated in HFD mice and further elevated by 39.3% in HFRD mice (Fig. 4D), suggesting that PPARγ may contribute to the development of NAFLD.

#### Riboflavin deficiency further impairs antioxidant function in mice fed the high fat diet

**Fig. 5:**
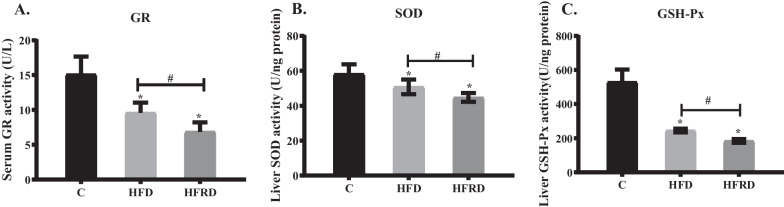
Changes of serum GR, liver SOD and GSH-Px activities after feeding different diets in mice. C57BL/6 mice were randomly divided into three groups, including control (C), high fat diet (HFD), high fat and riboflavin deficient diet (HFRD), and fed for 30 days. The activities of serum SOD **A**, liver GR **B** and GSH-Px **C** were determined using corresponding kits. Data are expressed as means ± SD (n = 12). *P < 0.05 versus C; #*P* < 0.05 versus HFD

GR, SOD and GSH-Px play critical roles in the antioxidant defense system [32–34] and their activities in the serum and liver were determined in the current study. Serum activity of GR was significantly reduced in mice fed the high fat diet. Meanwhile, the activities of SOD, and GSH-Px in the liver were significantly decreased in the HFD mice (Fig. 5A–C). Riboflavin deficiency further declined the activities of serum GR, liver SOD and GSH-Px by 28.6%, 11.9%, and 24.7%, respectively, in mice fed the high fat diet. These results indicate that riboflavin deficiency further impairs antioxidant function in the HFD mice.

### Cell culture

#### Riboflavin deficiency and PA load synergistically alter lipid metabolism in HepG2 cells

**Fig. 6:**
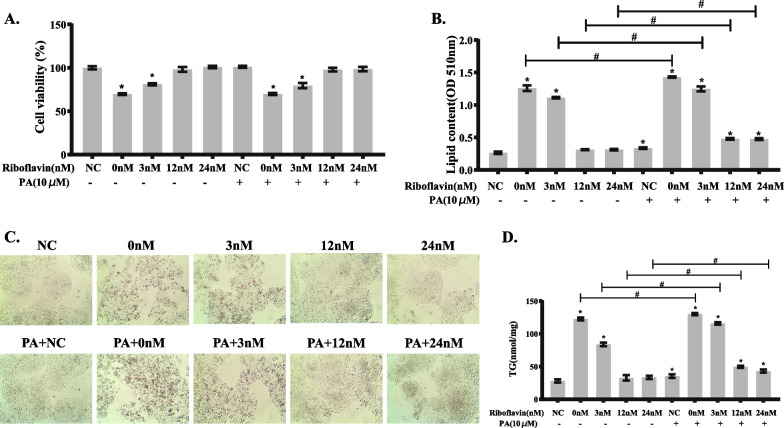
Synergistic action between riboflavin deficiency and PA load in increasing lipid accumulation in HepG2 cells. The experimental cells were cultured in media containing various concentrations of riboflavin (0, 3, 12, 24 nM) in the presence or absence of PA (10 μM) for 96 h. **A** Cell viability was determined by CCK8 assay. **B** TG content in HepG2 cells was detected using commercial kit. **C** Oil red O staining of HepG2 cells (40× magnification). **D** Quantitative determination of TG accumulation in HepG2 cells by a colorimetric method. Data are shown as mean ± SD, and experiments were performed in triplicate. *P < 0.05 versus NC; Comparisons between various concentrations of riboflavin (0, 3, 12, 24 nM) in the presence of PA (10 μM) versus the absence of PA were statistically significant at #*P* < 0.05

As shown in Fig. 6A, it was confirmed that 10 μM PA load did not significantly affect the viability of HepG2 cells. On the other hand, the viability of HepG2 cells was significantly reduced when being cultured in media containing 0 or 3 nM riboflavin. Cell viability was normalized when the concentration of riboflavin in the medium was over 12 nM. However, no significant interaction between riboflavin and PA was found on the viability of HepG2 cells (*P* > 0.05) (Additional file 2: Table S1).

As shown by Oil red O staining, the accumulation of lipid droplets in HepG2 cells treated with riboflavin deficiency or PA was higher than that in the control group. More lipid droplet accumulation was noted when HepG2 cells were treated with both riboflavin deficiency and PA load (Fig. 6B–C). The results of TG analysis also confirmed a significant increase in TG content in HepG2 cells after riboflavin deficiency or PA load compared to the control cells. More TG was detected in HepG2 cells when being exposed to the combination of riboflavin deficiency and PA load (Fig. 6D). Factorial analysis confirmed a significant interaction between riboflavin deficiency and PA load on TG accumulation in HepG2 cells (*P* < 0.05) (Additional file 2: Tables S2–3).

**Fig. 7:**
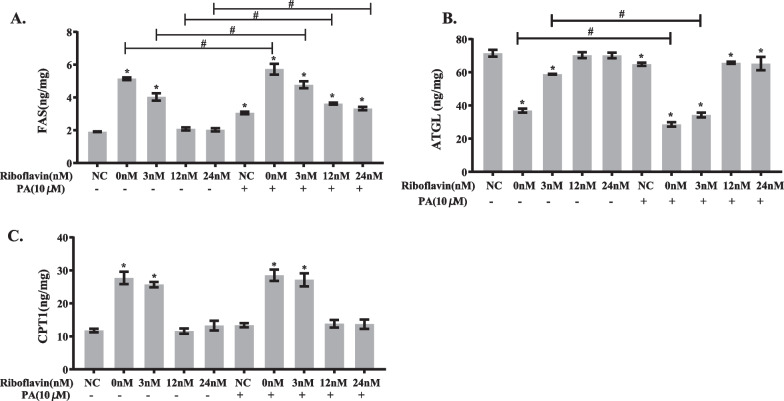
Synergistic action between riboflavin deficiency and PA load in altering the expression of lipid metabolism related enzymes in HepG2 cells. HepG2 cells were cultured in media containing various concentrations of riboflavin (0, 3, 12, 24 nM) in the presence or absence of PA (10 μM) for 96 h. The protein levels of FAS (**A**), ATGL (**B**) and CPT1 (**C**) were analyzed by ELISA methods. Data are shown as mean ± SD, and experiments were performed in triplicate. *P < 0.05 versus NC; Comparisons between various concentrations of riboflavin (0, 3, 12, 24 nM) in the presence of PA (10 μM) versus the absence of PA were statistically significant at #*P* < 0.05

To explore the effects of riboflavin deficiency combined with PA load on the protein expression of the enzymes related to lipid metabolism, intracellular contents of FAS, ATGL and CPT1 were measured using ELISA technique. The protein expression of FAS was significantly increased in HepG2 cells treated with riboflavin deficiency or PA load compared to the control cells (Fig. 7A). When cells were cultured in media containing 0 nM or 3 nM riboflavin in the presence of PA, FAS expression was further enhanced. A significant interaction occurred between riboflavin deficiency and PA load (*P* < 0.05) as shown by factorial analysis (Additional file 2: Table S4). As indicated in Fig. 7B, ATGL expression was significantly reduced in response to riboflavin deficiency or PA load compared to the control cells. ATGL expression was further declined when cells were cultured in media containing 0 nM or 3 nM riboflavin in the presence of PA. Factorial analysis demonstrated a synergistic action between riboflavin deficiency and PA load (*P* < 0.05) (Additional file 2: Table S5). PA load did not significantly affect the protein expression of CPT1 in HepG2 cells (Fig. 7C). However, riboflavin deficiency significantly increased the protein content of CPT1 at the concentration of 0 nM or 3 nM compared to the control cells. When cells were treated jointly with riboflavin deficiency and PA load, no further increase was noted in the intracellular CPT1 level. Factorial analysis did not demonstrate a significant interaction between riboflavin deficiency and PA load (*P* > 0.05) (Additional file 2: Table S6).

#### Riboflavin deficiency and PA load synergistically reduce antioxidant capacity in HepG2 cells

**Fig. 8:**
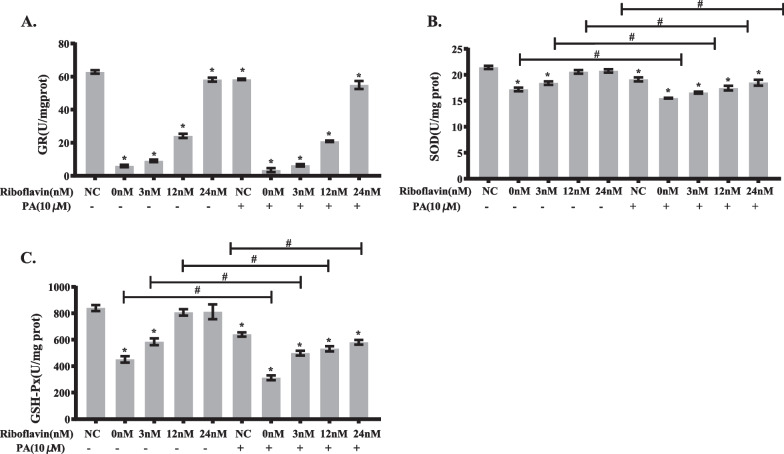
Synergistic action between riboflavin deficiency and PA load in reducing the activities of antioxidant enzymes in HepG2 cells. HepG2 cells were cultured in media containing various concentrations of riboflavin (0, 3, 12, 24 nM) in the presence or absence of PA (10 μM) for 96 h. The activities of GR (**A**), SOD (**B**), and GSH-Px (**C**) in HepG2 cells were determined using corresponding kits. Data are shown as mean ± SD, and experiments were performed in triplicate. *P < 0.05 versus NC; Comparisons between various concentrations of riboflavin (0, 3, 12, 24 nM) in the presence of PA (10 μM) versus the absence of PA were statistically significant at #*P* < 0.05

As indicated in Fig. 8A, GR activity was significantly lower in HepG2 cells treated with PA load or riboflavin deficiency than in the control cells. Although there was a further decline in GR activity in HepG2 cells when treated with PA load combined with riboflavin deficiency, the difference was not statistically significant. The activities of SOD and GSH-Px were also significantly reduced in riboflavin deficient or PA loaded cells. When treated with both PA load and riboflavin deficiency, the activities of SOD and GSH-Px were further significantly decreased in HepG2 cells (Fig. 8B–C). There was a significant interaction between riboflavin deficiency and PA load on the activities of SOD and GSH-Px, as demonstrated by factorial analysis (*P* < 0.05) (Additional file 2: Tables S7–9). Taken together, riboflavin deficiency further impairs antioxidant function in PA loaded HepG2 cells.

#### Synergistic effects of riboflavin deficiency and PA load on lipid metabolism are partly reversed by PPARγ gene silencing in HepG2 cells

**Fig. 9:**
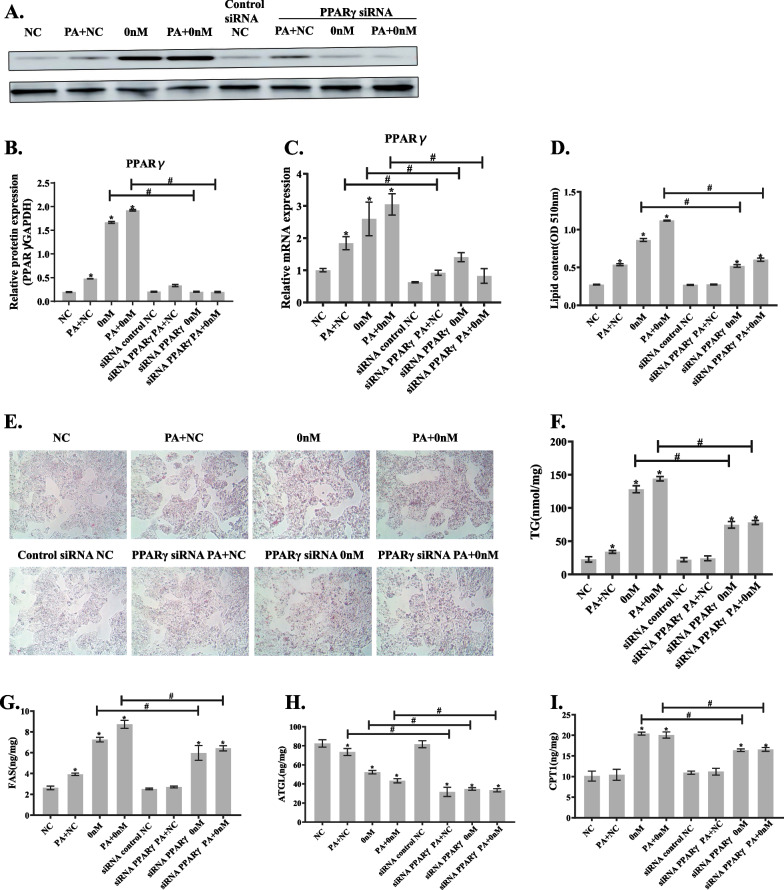
PPARγ gene silencing partly reverses disturbed lipid metabolism caused by riboflavin deficiency or/and PA load in HepG2 cells. HepG2 cells were cultured in the riboflavin free medium for 96 h in the presence or absence of PA (10 μM) after blockade of PPARγ expression with PPARγ small interfering RNA (siRNA). **A** PPARγ and GAPDH protein bands in HepG2 cells. **B** The histogram is the grey value analysis of the corresponding protein bands. **C** PPARγ mRNA expression in HepG2 cells. **D** Quantitative determination of lipid droplets in HepG2 cells. **E** Oil red O staining of HepG2 cells (40× magnification). **F** TG content in HepG2 cells were detected using a commercial kit. The protein levels of FAS **G**, ATGL **H **and CPT1 **I** in HepG2 cells were analyzed by ELISA assay. *P < 0.05 versus NC; Comparisons before and after PPARγ transfection were statistically significant at #*P* < 0.05

To explore the possible mechanisms related to the synergistic action between riboflavin deficiency and PA load on lipid metabolism, we transfected HepG2 cells with PPARγ siRNA and then cultured in PA loaded or/and riboflavin deficient media for 96 h. As seen in Fig. 9A–C, PPARγ protein and mRNA expressions were significantly decreased in HepG2 cells after PPARγ gene silencing, confirming a successful transfection. Meanwhile, lipid accumulation in the PA loaded cells was significantly decreased as evaluated by oil red O staining and TG assay (Fig. 9D–F). In the riboflavin deficient or riboflavin deficient and PA-loaded HepG2 cells, lipid droplet accumulation was significantly reduced after PPARγ gene silencing as showed by Oil red O staining. TG content was also significantly lower in HepG2 cells treated with riboflavin deficiency or riboflavin deficiency combined with PA load after PPARγ gene silencing by 230% and 284%, respectively as compared to the control cells. However, it did not recover completely to the normal level.

We further analyzed the changes of protein expressions of ATGL, FAS and CPT1 after PPARγ siRNA transfection (Fig. 9G–I). The results showed that the content of FAS in HepG2 cells treated with PA load returned to the normal level after PPARγ gene silencing. The expressions of FAS and CPT1 in HepG2 cells treated with riboflavin deficiency or riboflavin deficiency combined with PA load were also significantly lower after PPARγ siRNA transfection. The expression of FAS in riboflavin deficient HepG2 cells was decreased by 39% and that in riboflavin deficient and PA loaded HepG2 cells decreased by 77% after PPARγ silencing compared to the control cells. The expressions of CPT1 in HepG2 cells treated with riboflavin deficiency or riboflavin deficiency and PA load were decreased by 52% and 47%, respectively, after PPARγ gene silencing. We also found that the level of ATGL, an enzyme involved in TG hydrolysis, was significantly decreased in HepG2 cells treated with PA load or/and riboflavin deficiency after PPARγ silencing. These results indicate that the effects of riboflavin deficiency and PA load on lipid metabolism in HepG2 cells were alleviated after PPARγ gene silencing possibly by altering the expressions of the enzymes related to lipid metabolism.

## Discussion

In the early stage, NAFLD manifests as increaseing lipid accumulation in the liver. When the lipid content in the liver exceeds a certain threshold, hepatic steatosis will come into place [35, 36]. In this study, we utilized a diet-induced animal model of NAFLD, in which a high fat diet containing 42% of calories from fat and 0.2% of cholesterol was provided. It was showed that hepatic TG content was significantly increased after high fat diet feeding in mice, which is consistent with the data reported previously [37, 38]. FAS plays a critical role in hepatic lipogenesis and it was estimated that approximately 26% of the fat in the liver is from de novo lipogenesis in NAFLD patients [39]. In this study, the expression of hepatic FAS was significantly increased in the liver of HFD mice. ATGL, an enzyme response for TG hydrolysis [40, 41], was downregulated in expression in the liver of HFD mice. Meanwhile, antioxidant function was compromised as manifested by decreased activities of GR, SOD and GSH-Px. These data indicate that lipid metabolism, as well as the antioxidant defense system, is disturbed significantly by high fat diet feeding in mice and the animal model of NAFLD was successfully developed. The present study provides evidence that riboflavin deficiency and high dietary fat act synergistically in disturbing lipid metabolism and antioxidant function. More lipid accumulation was found in the liver when mice were exposed to both riboflavin deficiency and high fat diet. The expressions of some enzymes related to lipid metabolism, including FAS, ATGL and CPT1, were also significantly altered. Meanwhile, the activities of serum GR, liver SOD and GSH-Px were reduced more significantly in mice exposed to both riboflavin deficiency and high dietary fat.

Since HepG2 cells retain most of the biochemical and morphological properties of human hepatocytes and have been applied extensively in studying hepatic lipid metabolism in association with NAFLD [42–44], a cellular model of NAFLD was successfully developed using PA load in HepG2 cells in this study to further investigate the interaction between riboflavin deficiency and PA load [21, 45]. Factorial analysis confirmed a significant interaction between riboflavin deficiency and PA load on TG content and the expressions of FAS, ATGL and CPT1 in HepG2 cells, which is basically in line with the data obtained from the animal experiment. It was also noted that riboflavin deficiency led to a more significant drop in the activities of SOD and GSH-Px in PA exposed HepG2 cells.

PPARγ is one of the nuclear receptors expressed in adipose tissue, and regulates the expression of genes related to glucose and lipid metabolism [46]. Activation of the PPARγ gene in the liver increased de novo lipogenesis, and hepatocyte-specific PPARγ gene knockdown was associated with decreased de novo lipogenesis [47–49]. Under normal conditions, PPARγ expression is very low in the liver and increased with the development of hepatic steatosis in rodents and humans [7, 8, 50]. Currently, accumulated evidence supports the correlation between hepatic PPARγ expression and NAFLD development [51, 52]. Interestingly, Hino et al. reported that riboflavin deficiency impaired the function of the mitochondrial electron transport chain and activated the expression of PPARγ gene [53], implying that riboflavin deficiency may affect lipid metabolism by altering the expression of PPARγ gene. Our study found that riboflavin deficiency increased the expression of PPARγ in the liver of high fat diet fed mice. In vitro experiments also confirmed that the expression of PPARγ in HepG2 cells treated with PA load combined with riboflavin deficiency was significantly higher compared to those treated with PA load or riboflavin deficiency alone. After silencing the expression of PPARγ gene with corresponding siRNA, TG content was significantly decreased in HepG2 cells exposed to PA load or/and riboflavin deficiency. Furthermore, the expressions of FAS and CPT1 were changed toward to the normal. Therefore, PPARγ may play an important role in the combined actions of riboflavin deficiency and high dietary fat in the development of NAFLD by normalizing the expressions of the lipid metabolism related enzymes.

## Conclusions

We demonstrate for the first time that riboflavin deficiency and high dietary fat act synergistically to increase hepatic lipid accumulation both in vivo and in vitro, in which the PPARγ pathway may be significantly involved. Our study provides experimental evidence in supporting the “multiple hit” hypothesis that multiple insults, including high dietary fat and riboflavin deficiency, are interacted in contributing to the development of NAFLD. Further intervention studies in NAFLD patients with riboflavin malnutrition are needed to be carried out to further confirm the interaction between riboflavin deficiency and high dietary fat.

### Supplementary Information

**Additional file 1**. Feed composition in **Table S1** control, **Table S2** HFD and Table S3 HFRD group.

**Additional file 2**. Factorial analysis of the synergistic effect of riboflavin deficiency and palmitic acid in **Table S1** Cells activity, **Table S2** Oil red O staining, **Table S3** Triglyceride Levels, **Table S4** FAS protein levels, **Table S5** ATGL protein levels, **Table S6** CPT1 protein levels, **Table S7** GR activity, **Table S8** GSH-Px activity, **Table S9** SOD activity.

## Data Availability

The datasets used and/or analyzed during the current study are available from the corresponding author on reasonable request.

## Abbreviations

NAFLD: Non-alcoholic fatty liver disease
HFD: High-fat diet
PPARγ: Peroxisome proliferator-activated receptor gamma
ATGL: Adipose triglyceride lipase
ACAD: Acyl CoA dehydrogenase
FAS: Fatty acid synthase
CPT1: Carnitine palmitoyltransferase 1
NASH: Nonalcoholic steatohepatitis
FMN: Flavin mononucleotide
FAD: Flavin adenine dinucleotide
GR: Glutathione reductase
PA: Palmitic acid
DMSO: Dimethyl sulfoxide
BSA: Bovine serum albumin
ALT: Alanine aminotransferase
AST: Aspartate aminotransferase
TG: Triglycerides
TC: Total cholesterol
SOD: Superoxide dismutase
GSH-Px: Glutathione peroxidase
C: Control
HFRD: High-fat riboflavin-deficient diet
ELISA: Enzyme immunosorbent assay
NAS: NAFLD activity score
RT-PCR: Reverse transcription-polymerase chain reaction
siRNA: Small interfering RNA
