# Supplementary material for: Effects of riboflavin deficiency and high dietary fat on hepatic lipid accumulation: a synergetic action in the development of non-alcoholic fatty liver disease
Source: Nutr Metab (Lond). 2024 Jan 2;21:1. doi: 10.1186/s12986-023-00775-8 (PMC10763341; doi:10.1186/s12986-023-00775-8)
Supplement: Supplementary file 1 — Additional file 1. Feed composition in Table S1 control, Table S2 HFD and Table S3 HFRD group. [file 12986_2023_775_MOESM1_ESM.pdf]

Supplementary Material

Riboflavin deficiency and fatty acids synergistically increase hepatocyte lipid accumulation and oxidative stress

Table S1. Control feed formula

Composition	g/ kg
Casein	140.00
Cornstarch	465.69
Maltodextrin	155.00
Sucrose	100.00
Non-transgene soybean oil	40.00
Cellulose	50.00
Mineral mixture powder（AIN-93M-MX）	35.00
Vitamins mixture powder（AIN-93-MRDVX）	10.00
L-cystine	1.80
Choline chloride (50% choline)	2.50
Tertiary butylhydroquinone	0.01
Gelatin	30.00

Table S2. HFD feed formula

Composition	g/ kg
Casein	195.00
Cornstarch	150.00
Sucrose	344.16
Lard	210.00
Cholesterol	1.50
Cellulose	50.00
Mineral mixture powder（AIN-93M-MX）	35.00
Vitamins mixture powder（AIN-93-MRDVX）	10.00
L-cystine	1.80
Choline chloride	2.50
Tertiary butylhydroquinone	0.04
Gelatin	30.00

Table S3. HFRD feed formula

Composition	g/ kg
Riboflavin depleted casein	195.00
Riboflavin depleted cornstarch	150.00
Sucrose	344.16
Lard	210.00
Cholesterol	1.50
Cellulose	50.00
Mineral mixture powder（AIN-93M-MX）	35.00
Vitamins mixture powder without riboflavin （AIN-93-MRDVX）	10.00
L-cystine	1.80
Choline chloride	2.50
Tertiary butylhydroquinone	0.04
Gelatin	30.00
