# Supplementary material for: Effects of riboflavin deficiency and high dietary fat on hepatic lipid accumulation: a synergetic action in the development of non-alcoholic fatty liver disease
Source: Nutr Metab (Lond). 2024 Jan 2;21:1. doi: 10.1186/s12986-023-00775-8 (PMC10763341; doi:10.1186/s12986-023-00775-8)
Supplement: Supplementary file 2 — Additional file 2. Factorial analysis of the synergistic effect of riboflavin deficiency and palmitic acid in Table S1 Cells activity, Table S2 Oil red O staining, Table S3 Triglyceride Levels, Table S4 FAS protein levels, Table S5 ATGL protein levels, Table S6 CPT1 protein levels, Table S7 GR activity, Table S8 GSH-Px activity, Table S9 SOD activity. [file 12986_2023_775_MOESM2_ESM.pdf]

Supplementary Material

Riboflavin deficiency and fatty acids synergistically increase hepatocyte lipid accumulation and oxidative stress

1 Supplementary Tables

Factorial Analysis

Table S1 Cells activity

A. Tests of Normality

	Kolmogorov-Smirnov[a]			Shapiro-Wilk		
	Statistic	df	Sig.	Statistic	df	Sig.
NC	.211	3	.	.991	3	.817
Rib0	.358	3	.	.812	3	.144
Rib3	.175	3	.	1.000	3	1.000
Rib12	.306	3	.	.905	3	.401
Rib24	.190	3	.	.997	3	.903
PNC	.271	3	.	.947	3	.557
P0	.340	3	.	.848	3	.235
P3	.289	3	.	.927	3	.477
P12	.301	3	.	.912	3	.424
P24	.341	3	.	.847	3	.232

a. Lilliefors Significance Correction

B. Levene's Test of Equality of Error Variances[a]

Dependent Variable: CCK8

F	df1	df2	Sig.
1.392	9	20	.256

Tests the null hypothesis that the error variance of the dependent variable is equal across groups.

a. Design: + PA + Riboflavin + PA * Riboflavin

C. Tests of Between-Subjects Effects

Dependent Variable: CCK8

Source	Type III Sum of Squares	df	Mean Square	F	Sig.
Corrected Model	4669.927a	9	518.881	135.156	.000
	241100.210	1	241100.210	62800.723	.000
PA	2.249	1	2.249	.586	.453
Riboflavin	4656.487	4	1164.122	303.225	.000
PA * Riboflavin	11.192	4	2.798	.729	.583
Error	76.783	20	3.839		
Total	245846.920	30			
Corrected Total	4746.710	29			

a. R Squared = .984 (Adjusted R Squared = .977)

Table S2 Oil red O staining

A.Tests of Normality

	Kolmogorov-Smirnova			Shapiro-Wilk		
	Statistic	df	Sig.	Statistic	df	Sig.
NC	.198	3	.	.995	3	.870
Rib0	.360	3	.	.808	3	.134
Rib3	.215	3	.	.989	3	.800
Rib12	.184	3	.	.999	3	.927
Rib24	.229	3	.	.981	3	.739
PNC	.297	3	.	.917	3	.443
P0	.346	3	.	.837	3	.206
P3	.351	3	.	.826	3	.179
P12	.216	3	.	.989	3	.797
P24	.284	3	.	.934	3	.503

a. Lilliefors Significance Correction

B. Levene's Test of Equality of Error Variancesa

Dependent Variable: Oil Red O

F	df1	df2	Sig.
.910	9	20	.535

Tests the null hypothesis that the error variance of the dependent variable is equal across groups.

a. Design: + PA + Riboflavin + PA * Riboflavin

C.Tests of Between-Subjects Effects

Dependent Variable: Oil Red O

Source	Type III Sum of Squares	df	Mean Square	F	Sig.
Corrected Model	6.085a	9	.676	1523.911	.000
	15.689	1	15.689	35362.361	.000
PA	.151	1	.151	339.905	.000
Riboflavin	5.924	4	1.481	3337.889	.000
PA * Riboflavin	.011	4	.003	5.934	.003
Error	.009	20	.000		
Total	21.783	30			
Corrected Total	6.094	29			

a. R Squared = .999 (Adjusted R Squared = .998)

Table S3 Triglyceride Levels

A. Tests of Normality

	Kolmogorov-Smirnov[a]			Shapiro-Wilk		
	Statistic	df	Sig.	Statistic	df	Sig.
NC	.276	3	.	.942	3	.537
Rib0	.175	3	.	1.000	3	1.000
Rib3	.176	3	.	1.000	3	.988
Rib12	.219	3	.	.987	3	.780
Rib24	.253	3	.	.964	3	.637
PNC	.253	3	.	.965	3	.638
P0	.253	3	.	.964	3	.637
P3	.253	3	.	.964	3	.637
P12	.175	3	.	1.000	3	1.000
P24	.253	3	.	.964	3	.637

a. Lilliefors Significance Correction

B. Levene's Test of Equality of Error Variances[a]

Dependent Variable: TG

F	df1	df2	Sig.
.550	9	20	.820

Tests the null hypothesis that the error variance of the dependent variable is equal across groups.
a. Design: + PA + Riboflavin + PA * Riboflavin

C. Tests of Between-Subjects Effects

Dependent Variable: TG

Source	Type III Sum of Squares	df	Mean Square	F	Sig.
Corrected Model	54012.829[a]	9	6001.425	1021.176	.000
	174100.088	1	174100.088	29624.095	.000
PA	326.839	1	326.839	55.614	.000
Riboflavin	37872.573	4	9468.143	1611.057	.000
PA * Riboflavin	15813.416	4	3953.354	672.685	.000
Error	117.540	20	5.877		
Total	228230.457	30			
Corrected Total	54130.369	29			

a. R Squared = .998 (Adjusted R Squared = .997)

Table S4 FAS protein levels

A.Tests of Normality

	Kolmogorov-Smirnova			Shapiro-Wilk		
	Statistic	df	Sig.	Statistic	df	Sig.
NC	.175	3	.	1.000	3	1.000
Rib0	.177	3	.	1.000	3	.965
Rib3	.253	3	.	.965	3	.639
Rib12	.213	3	.	.990	3	.807
Rib24	.323	3	.	.878	3	.318
PNC	.187	3	.	.998	3	.916
P0	.332	3	.	.863	3	.275
P3	.215	3	.	.989	3	.797
P12	.353	3	.	.824	3	.173
P24	.209	3	.	.992	3	.825

a. Lilliefors Significance Correction

B.Levene's Test of Equality of Error Variancesa

Dependent Variable: FAS

F	df1	df2	Sig.
3.298	9	20	.013

Tests the null hypothesis that the error variance of the dependent variable is equal across groups.

a. Design: + PA + Riboflavin + PA * Riboflavin

C.Tests of Between-Subjects Effects

Dependent Variable: FAS

Source	Type III Sum of Squares	df	Mean Square	F	Sig.
Corrected Model	49.483a	9	5.498	221.639	.000
	382.598	1	382.598	15423.176	.000
PA	8.475	1	8.475	341.638	.000
Riboflavin	40.063	4	10.016	403.747	.000
PA * Riboflavin	.946	4	.236	9.531	.000
Error	.496	20	.025		
Total	432.578	30			
Corrected Total	49.979	29			

a. R Squared = .990 (Adjusted R Squared = .986)

Table S4 ATGL protein levels

A.Tests of Normality

	Kolmogorov-Smirnova			Shapiro-Wilk		
	Statistic	df	Sig.	Statistic	df	Sig.
NC	.193	3	.	.997	3	.889
Rib0	.232	3	.	.980	3	.726
Rib3	.276	3	.	.942	3	.537
Rib12	.248	3	.	.968	3	.657
Rib24	.194	3	.	.997	3	.888
PNC	.301	3	.	.912	3	.424
P0	.237	3	.	.977	3	.706
P3	.279	3	.	.939	3	.525
P12	.276	3	.	.942	3	.537
P24	.313	3	.	.894	3	.366

a. Lilliefors Significance Correction

B.Levene's Test of Equality of Error Variancesa

Dependent Variable: ATGL

F	df1	df2	Sig.
2.771	9	20	.028

Tests the null hypothesis that the error variance of the dependent variable is equal across groups.
a. Design: + PA + Riboflavin + PA * Riboflavin

C.Tests of Between-Subjects Effects

Dependent Variable: ATGL

Source	Type III Sum of Squares	df	Mean Square	F	Sig.
Corrected Model	7468.380a	9	829.820	250.636	.000
	96201.828	1	96201.828	29056.445	.000
PA	719.361	1	719.361	217.273	.000
Riboflavin	6331.359	4	1582.840	478.075	.000
PA * Riboflavin	417.660	4	104.415	31.537	.000
Error	66.217	20	3.311		
Total	103736.425	30			
Corrected Total	7534.598	29			

a. R Squared = .991 (Adjusted R Squared = .987)

Table S6 CPT1 protein levels

A. Tests of Normality

	Kolmogorov-Smirnov[a]			Shapiro-Wilk		
	Statistic	df	Sig.	Statistic	df	Sig.
NC	.274	4	.	.863	4	.272
Rib0	.217	4	.	.948	4	.701
Rib3	.205	4	.	.986	4	.935
Rib12	.233	4	.	.971	4	.850
Rib24	.227	4	.	.945	4	.684
PNC	.149	4	.	.995	4	.983
P0	.157	4	.	.990	4	.959
P3	.142	4	.	1.000	4	1.000
P12	.237	4	.	.966	4	.818
P24	.245	4	.	.887	4	.368

a. Lilliefors Significance Correction

B. Levene's Test of Equality of Error Variances[a]

Dependent Variable: CPT1

F	df1	df2	Sig.
1.295	9	30	.280

Tests the null hypothesis that the error variance of the dependent variable is equal across groups.
a. Design: + PA + Riboflavin + PA * Riboflavin

C. Tests of Between-Subjects Effects

Dependent Variable: CPT1

Source	Type III Sum of Squares	df	Mean Square	F	Sig.
Corrected Model	816.188[a]	9	90.688	113.532	.000
	6007.416	1	6007.416	7520.689	.000
PA	15.502	1	15.502	19.407	.000
Riboflavin	797.885	4	199.471	249.718	.000
PA * Riboflavin	2.801	4	.700	.877	.490
Error	23.964	30	.799		
Total	6847.567	40			
Corrected Total	840.151	39			

a. R Squared = .971 (Adjusted R Squared = .963)

Table S7 GR Activity

A. Tests of Normality

	Kolmogorov-Smirnova			Shapiro-Wilk		
	Statistic	df	Sig.	Statistic	df	Sig.
NC	.357	3	.	.814	3	.149
Rib0	.224	3	.	.984	3	.761
Rib3	.322	3	.	.880	3	.324
Rib12	.354	3	.	.821	3	.165
Rib24	.333	3	.	.862	3	.274
PNC	.349	3	.	.831	3	.190
P0	.207	3	.	.992	3	.834
P3	.265	3	.	.953	3	.584
P12	.176	3	.	1.000	3	.977
P24	.370	3	.	.786	3	.081

a. Lilliefors Significance Correction

B.Levene's Test of Equality of Error Variancesa

Dependent Variable: GR

F	df1	df2	Sig.
1.198	9	20	.349

Tests the null hypothesis that the error variance of the dependent variable is equal across groups.
a. Design: + PA + Riboflavin + PA * Riboflavin

C.Tests of Between-Subjects Effects

Dependent Variable: GR

Source	Type III Sum of Squares	df	Mean Square	F	Sig.
Corrected Model	17080.882a	9	1897.876	1423.855	.000
	27743.715	1	27743.715	20814.332	.000
PA	79.079	1	79.079	59.328	.000
Riboflavin	16998.489	4	4249.622	3188.219	.000
PA * Riboflavin	3.314	4	.828	.622	.652
Error	26.658	20	1.333		
Total	44851.255	30			
Corrected Total	17107.541	29			

a. R Squared = .998 (Adjusted R Squared = .998)

Table S8 GSH-Px Activity

A. Tests of Normality

	Kolmogorov-Smirnova			Shapiro-Wilk		
	Statistic	df	Sig.	Statistic	df	Sig.
NC	.253	3	.	.964	3	.637
Rib0	.292	3	.	.923	3	.463
Rib3	.292	3	.	.923	3	.463
Rib12	.253	3	.	.964	3	.637
Rib24	.204	3	.	.993	3	.843
PNC	.175	3	.	1.000	3	1.000
P0	.253	3	.	.964	3	.637
P3	.253	3	.	.964	3	.637
P12	.175	3	.	1.000	3	1.000
P24	.175	3	.	1.000	3	1.000

a. Lilliefors Significance Correction

B. Levene's Test of Equality of Error Variancesa
Dependent Variable: GSH-Px

F	df1	df2	Sig.
1.152	9	20	.375

Tests the null hypothesis that the error variance of the dependent variable is equal across groups.
a. Design: + PA + Riboflavin + PA * Riboflavin

C. Tests of Between-Subjects Effects
Dependent Variable: GSH-Px

Source	Type III Sum of Squares	df	Mean Square	F	Sig.
Corrected Model	733578.522a	9	81508.725	117.024	.000
	10770919.602	1	10770919.602	15464.057	.000
PA	225990.745	1	225990.745	324.460	.000
Riboflavin	486429.886	4	121607.472	174.595	.000
PA * Riboflavin	21157.890	4	5289.472	7.594	.001
Error	13930.264	20	696.513		
Total	11518428.388	30			
Corrected Total	747508.785	29			

a. R Squared = .981 (Adjusted R Squared = .973)

Table S9 SOD Activity

A. Tests of Normality

	Kolmogorov-Smirnova			Shapiro-Wilk		
	Statistic	df	Sig.	Statistic	df	Sig.
NC	.253	3	.	.964	3	.637
Rib0	.292	3	.	.923	3	.463
Rib3	.292	3	.	.923	3	.463
Rib12	.253	3	.	.964	3	.637
Rib24	.204	3	.	.993	3	.843
PNC	.175	3	.	1.000	3	1.000
P0	.253	3	.	.964	3	.637
P3	.253	3	.	.964	3	.637
P12	.175	3	.	1.000	3	1.000
P24	.175	3	.	1.000	3	1.000

a. Lilliefors Significance Correction

B.Levene's Test of Equality of Error Variancesa

Dependent Variable: SOD

F	df1	df2	Sig.
.761	9	20	.652

Tests the null hypothesis that the error variance of the dependent variable is equal across groups.

a. Design: + PA + Riboflavin + PA * Riboflavin

C.Tests of Between-Subjects Effects

Dependent Variable: SOD

Source	Type III Sum of Squares	df	Mean Square	F	Sig.
Corrected Model	100.119a	9	11.124	86.875	.000
	10335.316	1	10335.316	80713.584	.000
PA	37.467	1	37.467	292.602	.000
Riboflavin	60.691	4	15.173	118.491	.000
PA * Riboflavin	1.960	4	.490	3.827	.018
Error	2.561	20	.128		
Total	10437.996	30			
Corrected Total	102.680	29			

a. R Squared = .975 (Adjusted R Squared = .964)
